# Standardization of Workflow and Flow Cytometry Panels for Quantitative Expression Profiling of Surface Antigens on Blood Leukocyte Subsets: An HCDM CDMaps Initiative

**DOI:** 10.3389/fimmu.2022.827898

**Published:** 2022-02-11

**Authors:** Daniela Kužílková, Joan Puñet-Ortiz, Pei M. Aui, Javier Fernández, Karel Fišer, Pablo Engel, Menno C. van Zelm, Tomáš Kalina

**Affiliations:** ^1^ Childhood Leukaemia Investigation Prague (CLIP), Department of Paediatric Haematology and Oncology, Second Faculty of Medicine, Charles University, Prague, Czech Republic and University Hospital Motol, Prague, Czechia; ^2^ Department of Biomedical Sciences, University of Barcelona, Barcelona, Spain; ^3^ Department of Immunology and Pathology, Central Clinical School, Monash University, Melbourne, VIC, Australia; ^4^ Department of Allergy, Immunology and Respiratory Medicine, Central Clinical School, Monash University and Alfred Hospital, Melbourne, VIC, Australia

**Keywords:** flow cytometry, cluster of differentiation (CD), expression profiling, surfaceome, CD marker

## Abstract

**Background:**

The Human Cell Differentiation Molecules (HCDM) organizes Human Leukocyte Differentiation Antigen (HLDA) workshops to test and name clusters of antibodies that react with a specific antigen. These cluster of differentiation (CD) markers have provided the scientific community with validated antibody clones, consistent naming of targets and reproducible identification of leukocyte subsets. Still, quantitative CD marker expression profiles and benchmarking of reagents at the single-cell level are currently lacking.

**Objective:**

To develop a flow cytometric procedure for quantitative expression profiling of surface antigens on blood leukocyte subsets that is standardized across multiple research laboratories.

**Methods:**

A high content framework to evaluate the titration and reactivity of Phycoerythrin (PE)-conjugated monoclonal antibodies (mAbs) was created. Two flow cytometry panels were designed: an innate cell tube for granulocytes, dendritic cells, monocytes, NK cells and innate lymphoid cells (12-color) and an adaptive lymphocyte tube for naive and memory B and T cells, including TCRγδ^+^, regulatory-T and follicular helper T cells (11-color). The potential of these 2 panels was demonstrated *via* expression profiling of selected CD markers detected by PE-conjugated antibodies and evaluated using 561 nm excitation.

**Results:**

Using automated data annotation and dried backbone reagents, we reached a robust workflow amenable to processing hundreds of measurements in each experiment in a 96-well plate format. The immunophenotyping panels enabled discrimination of 27 leukocyte subsets and quantitative detection of the expression of PE-conjugated CD markers of interest that could quantify protein expression above 400 units of antibody binding capacity. Expression profiling of 4 selected CD markers (CD11b, CD31, CD38, CD40) showed high reproducibility across centers, as well as the capacity to benchmark unique clones directed toward the same CD3 antigen.

**Conclusion:**

We optimized a procedure for quantitative expression profiling of surface antigens on blood leukocyte subsets. The workflow, bioinformatics pipeline and optimized flow panels enable the following: 1) mapping the expression patterns of HLDA-approved mAb clones to CD markers; 2) benchmarking new antibody clones to established CD markers; 3) defining new clusters of differentiation in future HLDA workshops.

## Introduction

Since the development of hybridoma technology in 1975 (1), monoclonal antibody (mAb) production has been instrumental in examining protein expression and delineate cell types. Following its wide adoption, the need for quality assessment of antibody clones and consistency in naming their reactivity was quickly recognized, leading to the initiative of the Human Leukocyte Differentiation Antigen (HLDA) workshops (2, 3). Currently organized by the Human Cell Differentiation Molecules (HCDM), these wet-lab workshops have been run since the 1980s for experimental validation of the reactivity and specificity of mAb clones (2). Two or more validated clones recognizing the same protein target were clustered and designated a cluster of differentiation (CD) number (3). To date, ~400 targets have been assigned CD nomenclature, which ranges from CD1 to CD372 (4).

Flow cytometry is undoubtedly one of the key methods in which mAbs have been applied to evaluate protein expression in single cells (5). Multiparametric applications have expanded our knowledge in immunology and related fields, where the combinatorial expression of surface proteins identifies a particular cell type (6). At the same time, immunophenotyping has become a key method to diagnose hematological malignancies, performing disease classification (7) and associating the expression of particular markers with underlying leukemogenic molecular changes (8, 9).

HLDA workshop reports provide basic information on the reactivity of mAbs. However, these reports have been completed sequentially over 3 decades, scattering the expression information over many publications with a generally low number of investigated subsets (4, 10–14). Thus, a catalog containing comprehensive, quantitative and searchable CD marker expression data was missing until the CD Maps pilot project was published by the HCDM organization (15). Although this pilot project demonstrated the feasibility of a standardized and reproducible collection of the expression patterns, aspects of the procedures still required further optimization and conceptually different approaches, enabling the large-scale deployment and continual updatability of the CD Maps resource.

The construction of a comprehensive resource of CD marker expression should ideally include appropriate and assay-specific titration of each mAb reagent to use the optimal concentration for accurate molecule quantification and limit undesired background staining. In addition to standardized experimental procedures that are reproducible in time and place, the resource should be updatable and handle challenges with data management and annotations. Ultimately, a comprehensive combination of backbone markers is required to define the many functionally defined immune cell subsets in blood.

Although mAbs recognizing the same protein and showing similar reactivity patterns were clustered in CD workshops and assigned CD nomenclature, mAb clone performance may differ, making particular clones better suited for particular applications (16). These mAb clone differences can be defined by direct comparison in a standardized workflow, providing critical information to select the appropriate reagent for clinical studies - e.g., multisite cohort studies that must combine data analyses (17, 18).

Here, we developed a standardized and semi-automated procedure for high-throughput expression profiling of surface protein expression. We evaluated the standardization and optimization of high-throughput reagent titration, the polychromatic panel design for innate and adaptive blood immune cells and a bioinformatics pipeline for data analysis. This approach was validated globally across multiple centers with HLDA-approved antibody clones to CD3, CD11b, CD31, CD38 and CD40 and demonstrates the feasibility of antibody reactivity benchmarking within this framework.

## Material and Equipment

### Human Blood Samples and Cell Lines

The use of blood samples from healthy adults was approved by the Human Ethics Committees of Monash University, the Motol University Hospital, and the University of Barcelona and was contingent on informed consent in accordance with the Declaration of Helsinki. Blood buffy coats were obtained from the local blood banks. In addition, 4 human cell lines were selected as representatives of the cell types expressing the molecules targeted by the 11^th^ HLDA workshop: Raji (B cell) (19), Jurkat (T cell) (20), THP-1 (monocyte) (21), U266 (plasma cell) (22) (American Tissue Culture Collection (ATCC), Rockville, MD, USA). The mouse pre-B cell line 300.19 (23) (ATCC) served as a universal negative control.

### Flow Cytometry Equipment

Data acquisition was performed at three different centers using LSR II and LSR Fortessa (BD Biosciences) instruments equipped with 405 nm, 488 nm, 561 nm and 647 nm excitation lasers and a High Throughput Sampler (HTS).

## Methods

### Flow Cytometer Instrument Setup

Cytometer Setup and Tracking beads (BD Biosciences, San Jose, CA, USA) and 8-peak Rainbow bead calibration particles (Spherotech, Lake Forest, IL, USA) were used for PMT voltages and light scatter setup to achieve interlaboratory standardization as developed by the EuroFlow consortium (24). The PE-conjugated target mAbs were excited by the 561 nm laser; for each staining (well), a minimum of 0.5 million events were acquired. The EuroFlow Standard Operating Procedure (SOP) for Instrument Setup and Compensation can be downloaded from www.euroflow.org.

### Titration Procedure of Target mAbs

To accurately quantify expression, the target antibodies were PE-conjugated. The experimental setup for titrating large amounts of PE-conjugated antibodies was designed to be feasible at a large scale. To this end, a cellular mixture containing representatives of positive and negative cell subsets was created by mixing defined quantities (1x10^5^ of cells) of human peripheral blood cells and selected human (Raji, THP-1, Jurkat, U266) and mouse (300.19) cell lines. The cell lines were barcoded with cell tracking dyes as follows: mouse 300.19 and human U266 cell lines were stained with 20 and 5 µM CellTracker Blue CMHC Dye (Thermo Fisher Scientific, Waltham, Massachusetts, USA), respectively; human THP-1 and Jurkat cell lines were stained with 0.5 and 0.05 µM CellTracker Deep Red Dye (Thermo Fisher Scientific), respectively; human peripheral blood cells were stained with both 20 µM CellTracker Blue CMHC Dye and 0.5 µM CellTracker Deep Red Dye trackers. The human Raji cell line was left unstained. Cell tracker staining was performed according to the manufacturer’s protocols before mixing cells at equal rates and incubating them with PE-conjugated mAbs. The antibodies used were kindly provided by Exbio Praha, Vestec, Czech Republic (CD31, MEM-05; CD38, HIT2; CD3, UCHT1; CD3, SK7; CD3, TB3; CD3, MEM-57) and BioLegend, San Diego, California, USA (CD40, 5C3; CD11b, and ICRF44). All the mAbs were evaluated in with dilutions ranging from 1/5 to 1/3200 to determine the optimal titer at the edge of saturation. The dilution recommended by the manufacturer was chosen as the starting point.

### Computer-Assisted Experimental Protocol Setup

To enhance the ease of tracking and repeating the experimental procedure, we established an automated process for protocol preparation based on a manually completed “Experiment Master Table” (EMT) using R software (http://www.r-project.org/). Briefly, the EMT was prepared as an Excel table with information about the sample, backbone panel, antibodies, user and so on. Information in the EMT is used (via web front-end) to automatically generate an experimental protocol that is time-stamped and includes calculated amounts of master mixes and pipetting volumes for all wells in all plates. This setting minimizes user errors and allows the tracking and archiving of the complete procedure.

### Single Cell Isolation and Preparation

The blood leukocyte isolation protocol was optimized to minimize platelet adhesion (satellitism) (25). Briefly, the buffy coat suspension contained citrate phosphate dextrose as anticoagulant, and was diluted 6× in PBS containing 2 mM EDTA, followed by the addition of an equal volume of a 4% dextran solution (Sigma–Aldrich, Saint Louis, MO, USA) in 0.9% NaCl. The mixture was left for 30 min for erythrocytes to sediment before collecting the supernatant containing the leukocytes. Following spinning (670 g, 5 min, RT) and removal of the supernatant, the white blood cell count was adjusted to 5×10^7^/ml in PBS supplemented with 0.09% NaN_3_, 0.5% BSA and 20% rabbit serum (Biowest, Nuaillé, France).

### Staining of Blood Leukocytes for Expression Profiling

Cells were stained in 96-well Polypropylene DeepWell plates in a total suspension volume of 50 µl. First, each of the PE-labeled mAbs was added to each well (the marker details are listed in **Supplementary Table S1**). The mAb amounts were derived from the titration experiment, and PBS supplemented with 0.09% NaN_3_, 0.5% BSA and 20% rabbit serum was added to a final volume of 10 µl. Subsequently, 40 µl of leukocyte cell suspension (2 × 10^6^ cells) was added to each well. Following careful mixing, the suspensions were incubated for 30 min at room temperature (RT) in the dark. Next, 25 µl of backbone mAb reagent mix was added to each well. Following careful mixing, the plate was incubated for an additional 30 min (RT, in the dark). The compositions of the two backbone antibody panels (innate and adaptive) were optimized, and the reagents were titrated beforehand (the details are provided in **Table 1**). Most of the backbone reagents were custom provided in an mAb mix that was dried in 96-well plates as HLDA innate and HLDA adaptive panels within Dry Reagents (Exbio Praha, Vestec, Czech Republic), with polymer-conjugated mAbs (BioLegend, Inc., San Diego, CA, USA) added from a liquid stock. All the mAb conjugates were generously donated by Exbio and BioLegend. The residual erythrocytes were lysed by Excelyse Easy solution (Exbio) according to manufacturer´s instructions. Briefly, 1.5 ml of 10× diluted Excelyse Easy solution was added to the 75μl cell suspension incubated for 10 min at RT, in the dark. This procedure provided a mild fixation condition to preserve fragile subsets (e.g. T follicular helper cells - Tfh). The samples were centrifuged (670 g, 5 min, RT), supernatant was removed, and the pellet was dried with a wool pulp. The cell pellet was resuspended in 200 µl of PBS for acquisition and stored at 4°C overnight.

**Table 1:** Reagents used in the HLDA innate and HLDA adaptive panel.

Innate tube														
**Fluorochrome**	**BV421**	**Pac Orange**	**BV605**	**BV711**	**FITC**	**PE**	**PE-DyLight 594**		**PerCP-Cy5.5**	**PE-Cy™7**	**APC**	**Alexa Fl. 700**	**APC-Cy™7**	
Target	CD127	CD45	CRTH2	CD56	CD117	tested CD	CD3	CD19	CD14	CD11c	CD123	HLA-DR	CD16	
clone	A019D5	2D1	BM16	HCD56	104D2		UCHT1	LT19	MEM-15	BU15	6H6	L243	3G8	
Volume	1.25 µl	5 µl	2 µl	1.25 µl	2.5 µl		2.5 µl	2.5 µl	2.5 µl	1.25 µl	1.25 µl	2.5 µl	2.5 µl	
**Adaptive tube**														
**Fluorochrome**	**Pac Blue**	**Pac Orange**	**BV605**	**FITC**	**PE**	**PE-Dazzle594**	**PerCP-Cy5.5**		**PE-Cy™7**		**APC**	**Alexa Fl. 700**	**APC-Cy™7**	
Target	CD45RA	CD45	CXCR5	CD27	tested CD	CD127	CD4	IgD	TCRgd	CD19	CD25	CD3	CD8	
clone	MEM-56	2D1	J252D4	LT27		A019D5	MEM-241	IA6-2	B1	LT19	MEM-181	UCHT1	MEM-31	
Volume	5 µl	5 µl	0.625 µl	2.5 µl		0.625 µl	2.5 µl	2.5 µl	5 µl	1.25 µl	1.25 µl	2.5 µl	2.5 µl	dried reagents

Dried reagents shaded.

### Automated FCS File Check and Annotation

All acquisitions were performed using default cytometer acquisition software settings for FCS file labeling (e.g., Specimen_001_A1_A01_001.fcs). The previously prepared EMT table was used for automated renaming of FCS files and their FCS header fields to include all relevant experimental information from the EMT table. This facilitated automated and standardized annotation of FCS files for further analysis.

### Conversion of PE Fluorescence Intensity to Antibody Binding Capacity (ABC)

PE conjugation of mAbs is consistent with a 1:1 ratio of fluorochrome:antibody, facilitating the calculation of the antibody binding capacity (ABC) from PE fluorescence. To convert PE fluorescence to the amount of PE molecules bound to a target, we used the PE Fluorescence Quantitation Kit (BD Biosciences) with four known levels of PE. The pellet was resuspended in 500 μL of PBS supplemented with 0.09% NaN_3_ plus 0.5% BSA and analyzed by flow cytometry in parallel with each experiment. The measured PE signals for all stainings on all cell subsets were fitted to the PE calibration curve to extract the number of PE molecules as described previously (15).

### Analysis, Gating and Export of Values

The leukocyte and lymphocyte subsets to be analyzed were predefined and gated uniformly by a single operator using FlowJo (v10, BD Biosciences). From each defined subset, the median intensity in the PE channel and median intensity of ABC were extracted. For each subset, the 90^th^ percentile of the ABC value on empty PE channels (fluorescence minus one; FMO) was considered the cutoff for the subset-specific background. The interquartile range (IQR) was calculated as IQR = Q3 − Q1. The minimum cell count for statistical evaluation was set to 66, and subsets with lower cell counts were omitted from further analysis. The FCS data and the FlowJo workspaces are deposited on the HCDM website (https://www.hcdm.org/index.php/2016-12-06-21-38-08/cdmaps-data-repository).

## Results

### Automated Workflow for High-Throughput Expression Profiling

**Figure 1:**
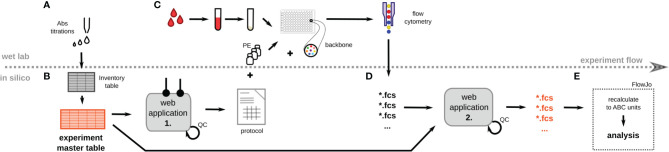
Schematic overview of the CD Maps workflow. **(A)** All Phycoerythrin (PE)-conjugated monoclonal antibodies (mAbs) were titrated using mixture of cell lines and peripheral blood leukocytes. Inventory table with all relevant details of individual clones was created. **(B)** The inventory table serves as a template for the experiment master table (EMT) which lists the details of a particular experiment, e.g. the position of individual mAbs in 96-well plate, experiment name, operator etc. Based on the EMT, an experimental protocol is created with automated calculation of reagent amounts and volumes. **(C)** Peripheral blood leukocytes were isolated, stained with PE-labeled and backbone mAbs, and acquired on a flow cytometer using the High Throughput Sampler (HTS). Quantibrite-PE beads are acquired in parallel with each experiment. **(D)** After acquisition and export of fcs files, these were uploaded for online annotation with details from the EMT (such as CD name, gene name, clone, experimental details etc.) introduced as new keywords into each fcs file. **(E)** The antibody binding capacity (ABC) of each PE marker is calculated on the basis of the Quantibrite-PE bead signal from the PE channel. Defined leukocyte subsets are gated using a pre-defined template for evaluation of expression levels of the PE marker. QC, quality control.

The scale of the intended CD Maps project required the following: 1) the processing of hundreds of measurements a day; 2) interlaboratory collaboration; 3) reproducibility in time and place. Thus, we designed a structure for high-throughput experiment execution (**Figure 1**) sourcing the EMT of PE reagents to be tested. The EMT contained all identifiers of a reagent (clone name, origin, CD name, gene name) (**Supplementary Table S1**).

A SOP for leukocyte isolation and antibody labeling in a 96-well plate format was developed, and a website interface was made that allowed for the creation of customized experimental protocols using the EMT and SOP in a printable format (**Data Sheet 1**). Thus, the position of each PE reagent was assigned to a well of the 96-well plate, printed out and the EMT tables represented the history of the processed experiments.

Dried reagent cocktails in a 96-well plate format were designed, titrated and custom produced to ensure speed, precision and stability of the backbone reagents used for universal gating. After acquisition on the HTS, proper compensation of the PE channel was verified using FMO control wells, and the files were exported as FCS 3.0. Next, the batch of FCS files was processed using a website interface that annotated the FCS files with the set of reagent identifiers from the EMT. Finally, this workflow generated fully annotated FCS files with consistent reagent identifiers, allowing subsequent batch analysis (**Data Sheet 2**).

### Strategy for the Titration of PE Reagents

**Figure 2:**
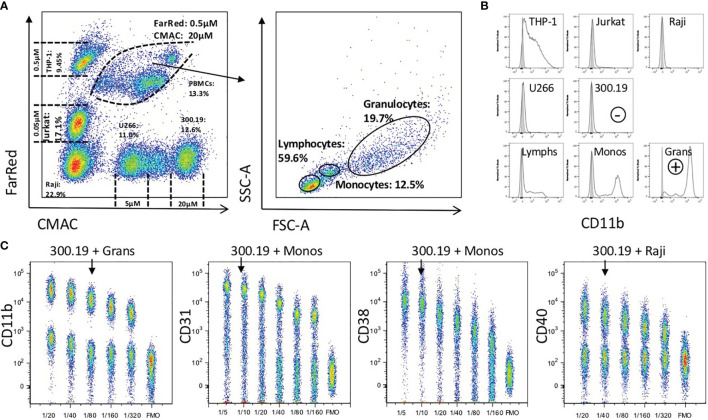
Universal titration procedure for PE-labeled mAbs. **(A)** Five cell lines (Raji, Jurkat, THP-1, U266 and 300.19) and fresh peripheral blood leukocytes were barcoded using Cell Tracker Dyes (CMAC and Far Red), mixed together and further stained with different amount of PE-labeled mAbs. Lymphocytes, monocytes and granulocytes from peripheral blood were gated based on their FSC and SSC. **(B)** Histograms show intensity of CD11b-PE (black histogram) and unstained (grey-filled histogram) on gated cell types. Selection of mouse cell line 300.19 as negative (-) and granulocytes as positive (+) cell type is shown **(C)** Titration plots for CD11b, CD31, CD38 and CD40 on a positive and negative cell type are shown (cell type used is shown above each dot plot). The X-axis depicts the dilution of the indicated PE-labeled mAb, The Y-axis represents the PE signal intensity. A fluorescence minus one (FMO) control is included in each titration plot. The titer of each mAb that was selected for expression profiling is indicated with an arrow.

For accurate quantification of protein expression in ABC, all the PE reagents must be optimally titrated (5). To optimize the procedure for high-throughput processing, a uniform titration protocol was developed using a mixture of defined cell lines corresponding to B and T lymphocytes, plasma cells and monocytes, and fresh human peripheral blood cells. Each cell line was uniquely barcoded with a combination of three intensities of two cell tracking dyes, and their combination ensured that for nearly all reagents, a positive and a negative population was present in a single tube (**Figure 2**). Individual cell types were identified and electronically gated based on the differential cell tracking dye and light scatter characteristics (**Figure 2A**), followed by manual selection of positive and negative cell types to evaluate the optimal titer (**Figure 2B**). The murine cell line 300.19 was used as a universal negative control for the anti-human antibodies. The optimal mAb titer was defined using the following criteria: the positive cell type yielded near maximal intensity (near saturation), and the negative control cell line showed minimal signal background. While titration of CD31, CD38 and CD40 showed a negligible background at the saturation titer, the optimal titer of CD11b was chosen below saturation to keep the background at a low level, reducing false-positive staining results (**Figure 2C**). Our approach prioritizing accuracy of expression level determination and low false positive cells in template gating was confirmed by Stain Index calculation [with modification by Telford (26)], prioritizing signal to noise resolution that yielded the same titer in three out of 8 mAbs (**Data Sheet 3**). In the other five mAbs we selected one step lower titer than at maximum Stain index in order to limit the false positive proportion of positive cells in the CD maps dataset, however the impact on resolution and on the expression level was negligible. Although in the case of CD31 and CD38 different intensities of expression are observed among peripheral blood subtypes (Granulocytes vs. Monocytes vs. Lymphocytes) optimal titers do not differ by using either subtype (**Data Sheet 4**).

### Backbone Panel Design and Performance Across Laboratories

Following completion of the CD Maps CD1-100 study (15), we identified the need to include additional blood lymphocyte subsets that are of major interest to clinical research and are considered relevant for diagnostics and disease monitoring. Thus, we adjusted the blood innate and adaptive tubes with extension of the fluorescent parameters for the backbone from 7 to 10-11 (**Table 1**). The innate cell tube was extended with CD117, CD127 and CRTH2 to facilitate the identification of ILC-1, ILC-2 and ILC-3 subsets (27, 28), bringing the total number of innate cell subsets to 12 (**Table 2**). The lymphoid tube was expanded with CD25, CD127 and CXCR5, enabling the detection of T regulatory cells (Tregs) (29) and Tfh cells (30, 31) with a total of 15 defined cell subsets (**Table 2**). The detailed gating strategies for the innate and adaptive cell tubes are shown in **Figures 3**, **4**.

**Table 2:** Definitions of the leukocyte subsets defined in this study.

Population name	Population code	Immunophenotype	Background cut-off (ABC units)
**Granulocytes**	**Granulocytes**	CD45+/CD3-/CD19-/	
Neutrophils	Neutrophils	SSC high/CD16+	408
Eosinophils	Eosinophils	SSC very high/CD16-	632
Basophils	Basophils	SSC low/CD123+/HLA-DR-	357
**Monocytes**	**Monocytes**	CD45+/CD3-/CD19-/SSC low/CD123-/HLA-DR+/CD11c+	
classical monocytes	class Mono	CD14+CD16-	384
intermediate monocytes	inter Mono	CD14+CD16+	353
nonclassical monocytes	nonc Mono	CD14-CD16+	350
**Dendritic cells**	**Dendritic cells**	CD45+/CD3-/CD19-/SSC low	
myeloid dendritic cells	mDC	CD123-/CD11c+/CD16-CD14-/HLA-DR++	396
plasmacytoid dendritic cells	pDC	CD123+ HLA-DR+	309
**Innate lymphoid cells**	**Innate lymphoid cells**	CD45+/CD3-/CD19-/SSC very low/CD123-/HLA-DR-/CD14-/CD127+/CD16-	
Innate lymphoid cells 1	ILC-1	CRTH2-CD117-	336
Innate lymphoid cells 2	ILC-2	CRTH2+CD117-	312
Innate lymphoid cells 3	ILC-3	CRTH2-CD117+	459
**NK cells**	**NK cells**	CD45+/CD3-/CD19-/SSC very low/CD123-/HLA-DR-/CD14-/CD127-/CD56+ and/or CD16+	229
**T cells**	**T cells**	CD45+/SSClow/CD3+/CD19-/	** **
TCRγδ+ T cells	Tgd	TCRgd+	461
**CD4 helper T cells**	**CD4**	CD4+/CD8-	
CD4 naive	CD4 naive	CD45RA+/CD27+	369
CD4 Central Memory	CD4 CM	CD45RA-/CD27+	471
CD4 Effector Memory	CD4 EM	CD45RA-/CD27-	406
CD4 CD45RA+ effector memory	CD4 TEMRA	CD45RA+/CD27-	360
Regulatory T cells	Treg	CD25+CD127-	305
Follicular helper T cells	Tfh	CD45RA-/CXCR5+	415
**CD8 cytotoxic T cells**	**CD8**	CD4-/CD8+	
CD8 naive	CD8 naive	CD45RA+/CD27+	384
CD8 Central Memory	CD8 CM	CD45RA-/CD27+	425
CD8 Effector Memory	CD8 EM	CD45RA-/CD27-	396
CD8 CD45RA+ effector memory	CD8 TEMRA	CD45RA+/CD27-	338
**B cells**	**B cells**	CD45+/SSClow/CD3-/CD19+	
B naive	B naive	IgD+/CD27-	773
B double negative	B dn	IgD-/CD27-	732
B nature Effector	B nat Eff	IgD+/CD27+	786
B switched Memory	B sw Mem	IgD-/CD27+	727

The background cut-off value was determined as the median of the 90 percentile FMO values from all analyzed donors.

**Figure 3:**
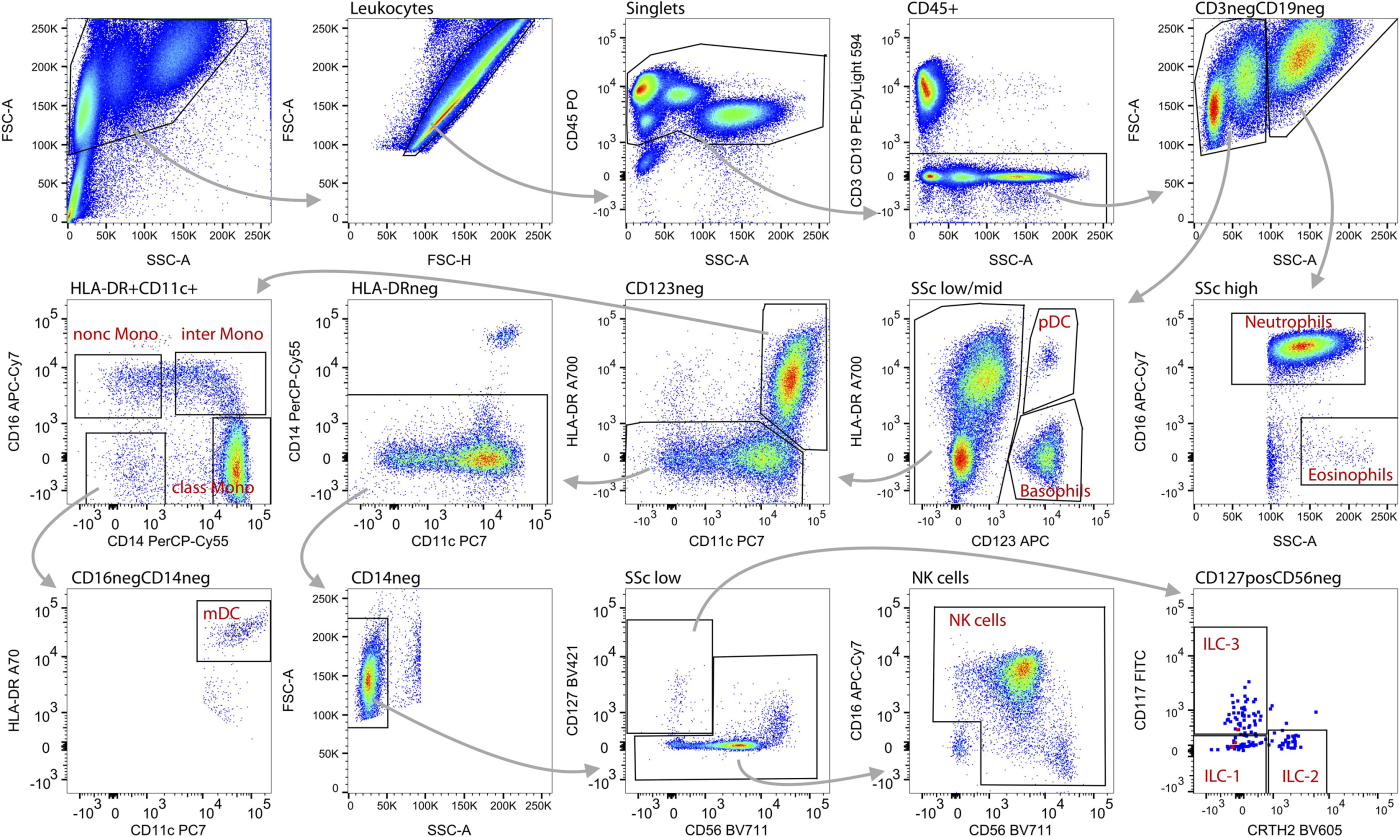
Gating strategy and identification of innate blood cell types. Within total acquired events, first the debris (low FCS) was excluded (top left), followed by doublets (non-linear events on FSC-A vs. FSC-H plot; 2^nd^ plot top row). Subsequently, CD45^neg^ events were removed (3^rd^ plot) as well as CD3 or CD19 expressing cells (4^th^ plot). Within these CD45^+^CD3^neg^CD19^neg^ innate blood cells, neutrophils were gated on the basis of SSc^high^CD16^+^ and eosinophils as SSc^high^CD16^dim^ events (5^th^ plot middle row). Within the SSc^low/med^ fraction,basophils were defined as SSc^low/med^CD123^+^HLA-DR^neg^ and plasmacytoid dendritic cells (pDC) as SSc^low/med^CD123^+^HLA-DR^+^ (4^th^ plot). Within CD123^neg^ myeloid dendritic cells (mDC) were gated as SSc^low/med^CD123^neg^CD14^neg^CD16^neg^CD11c^high^HLA-DR^high^ (middle panel, 3^rd^ and 1^st^ plot; bottom left plot). Monocytes were gated as SSc^low/med^CD123^neg^HLA-DR^+^CD11c^+^ (middle panel, 3^rd^ plot) and further divided into classical (CD14^+^CD16^neg^), intermediate (CD14^+^CD16^+^) and non-classical (CD14^neg^CD16^+^) phenotype (middle panel, left). Within the CD123^neg^ HLA-DR^neg^ (middle panel, 3^rd^ plot) CD14 expressing cells were excluded (middle panel, 2^nd^ plot) and lymphocytes were gated on the basis of SSC^low^ (bottom panel, 2^nd^ plot). Within the SSC^low^ fraction innate lymphoid cells (ILC) were identified as CD127^+^ (bottom panel, 3^rd^ plot) and further divided into ILC-1 (CD117^neg^CRTH2^neg^), ILC-2 (CD117^neg^CRTH2^+^) and ILC-3 (CD117^+^CRTH2^neg^) (bottom right). For gating of NK cells CD127^+^CD56^neg^ cells were excluded (bottom panel, 3^rd^ plot) and finally NK cells were identified as CD56^+^ and/or CD16^+^ (bottom panel, 4^th^ plot).

**Figure 4:**
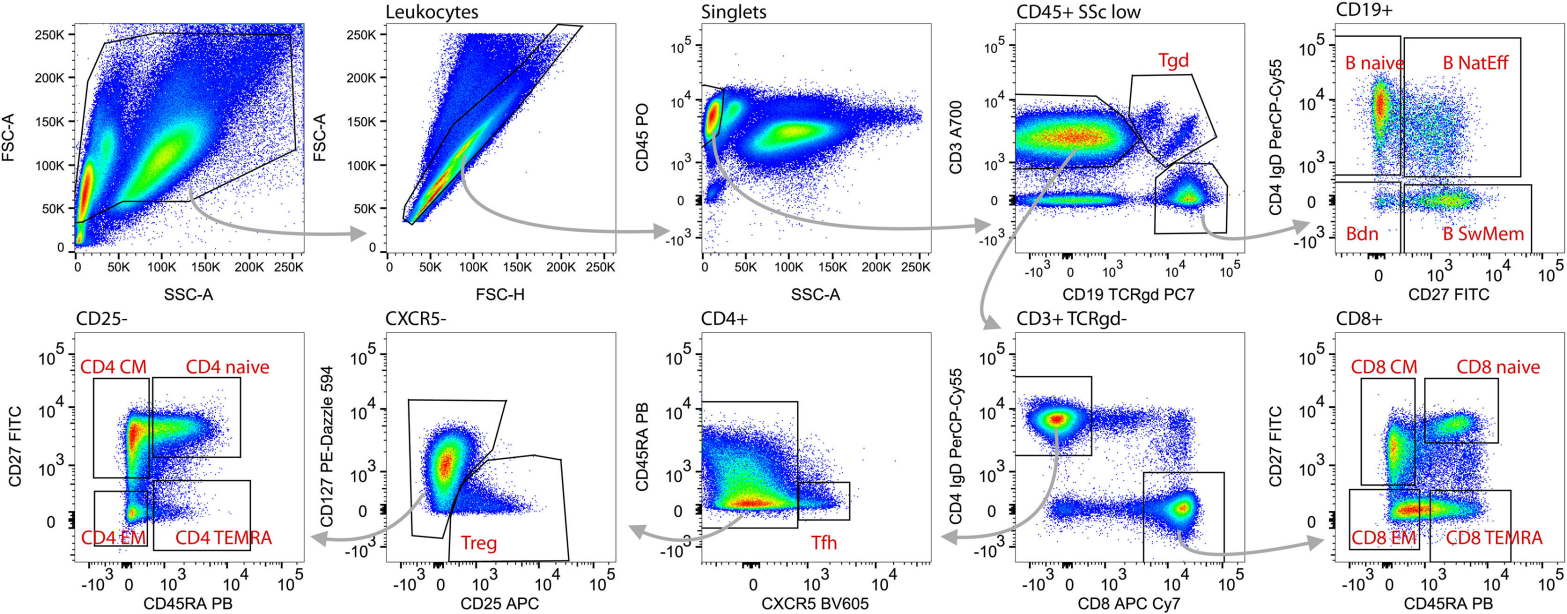
Gating strategy and identification of adaptive lymphocytes. Within total acquired events, first the debris (low FCS) was excluded (upper left plot) followed by doublets (non-linear events on FSC-A vs. FSC-H plot; 2^nd^ plot upper row). Lymphoid blood cell types were defined as CD45^hi^SSc^low^ (3^rd^ plot upper row). Gamma delta T cells (Tgd) were gated as T cell receptor (TCR) γδ^+^CD3^+^ (4^th^ plot upper row). B cells were identified as CD3^neg^CD19^+^ (4^th^ plot upper row) and subsequently divided into naive (B naive, CD27^neg^IgD^+^), nature effector B cells (B nat Eff, CD27^+^IgD^+^) switched memory B cells (B sw Mem, CD27^+^IgD^neg^) and double negative B cells (Bdn, CD27^neg^IgD^neg^; upper right plot). T cells were gated as CD3^+^CD19^neg^TCRγδ^-^ (4^th^ plot upper row) and on the basis of CD4 or CD8 expression divided into CD4 T helper cells (T CD4) and CD8 cytotoxic T cells (T CD8; 4^th^ plot lower row). Within the T CD4 cells follicular helper CD4 T cells (Tfh) were gated as CXCR5^+^CD45RA^neg^ (3^rd^ plot lower panel), regulatory T cells (Treg) as CD25^+^CD127^neg^ (2^nd^ plot lower panel). Remaining CD4 T cells (left plot lower row) as well as CD8 T cells (right plot lower row) were subdivided based on CD27 and CD45RA expression to naive (CD45RA^+^CD27^+^), Central Memory (CD45RA^neg^CD27^+^), Effector Memory (CD45RA^neg^CD27^neg^), and Terminal Effector Memory RA^+^ T cells (CD45RA^+^CD27^neg^).

In a previous study, autofluorescence and spreading error limited the sensitivity of the low PE signal because the 488 nm laser was used for measurement (15). Here, we sought to establish the lower limit of detection of the optimized panels using 561 nm laser excitation of PE. Advancing our study from the pilot using 8 fluorochromes (7 backbone + PE marker) to 11 or 12 with PE excitation with a 561 nm laser necessitated using 4 laser conventional flow cytometers. BD LSRII and BD Fortessa instruments were used with similar but not identical detection optics, where one instrument lacked the BV711 detection channel, and samples acquired on that analyzer were not stained with CD56-BV711 in the innate tube. In those samples, NK cells, ILC-2 and ILC-3 (but not ILC-1) were adequately resolved. The minimal ABC resolution was determined as the 90^th^ percentile of the ABC value on the FMO control tube (**Table 2**). The median level of the minimal ABC resolution across subsets was 396 ABC units (229 to 786, minimum to maximum), and this threshold was used to gate positive events for each evaluated reagent.

### Feasibility and Reproducibility of CD Maps Resource Building

The automated procedure and standardized experimental approach were evaluated by expression profiling of four HLDA-approved clones to CD markers. The four CD markers CD11b, CD31, CD38 and CD40 were selected based on their known distinct expression profiles across cell lineages: CD11b is expressed on myeloid cells (32), CD40 is expressed on B cells (33), and CD31 and CD38 show various degrees of expression across myeloid and lymphoid cell subsets (34, 35) but at different intensity levels.

**Figure 5:**
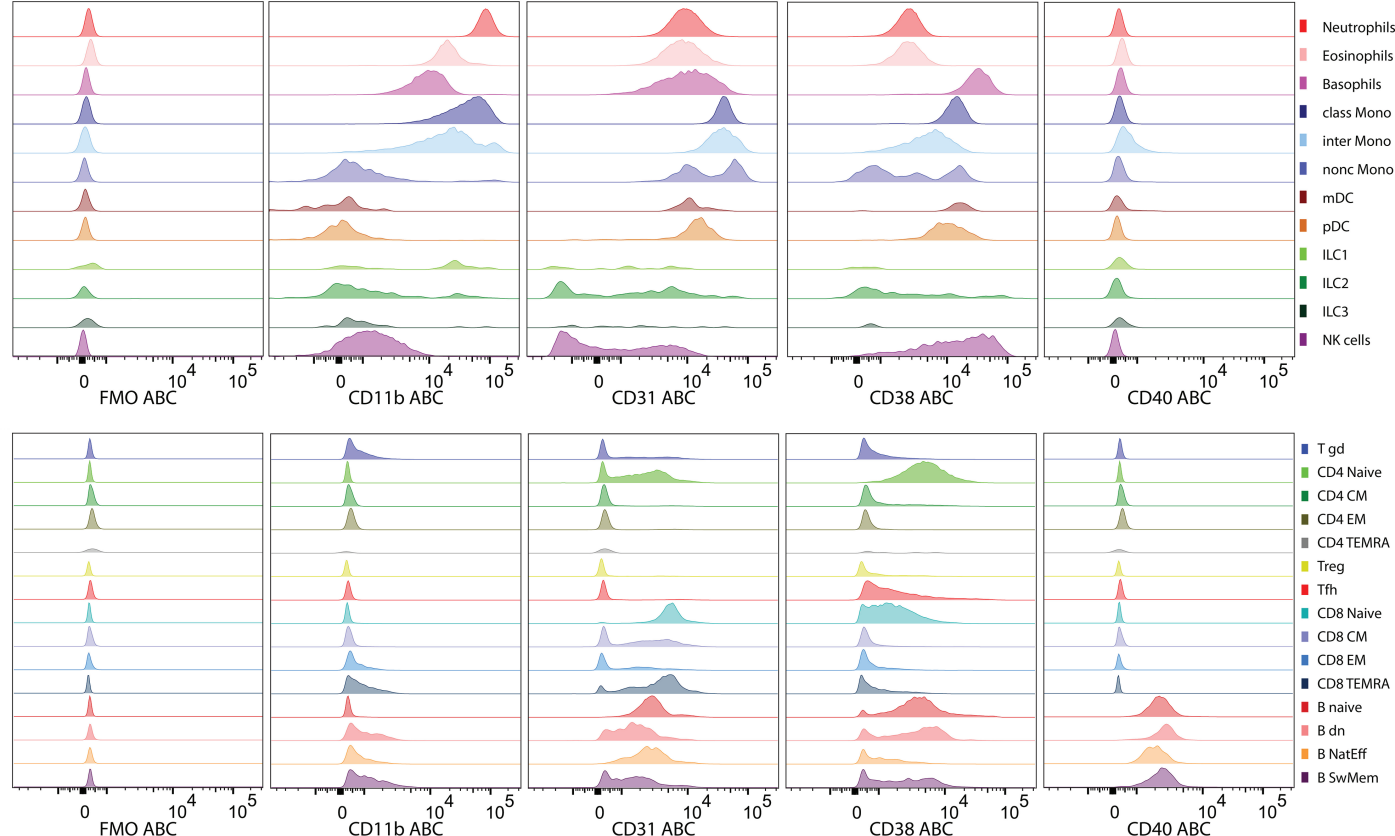
Levels of Antibody Binding Capacity (ABC) on leukocyte subsets. **(A)** Histograms of expression levels of CD11b, CD31, CD38 and CD40 are shown on innate leukocyte subsets and **(B)** on adaptive lymphocyte subsets of a representative subject in comparison to fluorescence minus one (FMO) controls. ABC units show expression levels recalculated from PE-antibody conjugates providing a standardized quantification.

CD11b expression was the highest (evaluated as median ABC) on neutrophils and classical monocytes but was moderately intensive on eosinophils, basophils and intermediate monocytes and lacking on nonclassical monocytes, dendritic cells, NK cells and adaptive lymphocytes (**Figure 5A**). CD31 and CD38 were expressed on naive CD4 and CD8 T cells. However, although CD38 was absent at later differentiation stages in steady-state T cells, CD31 was also expressed on memory CD8 T cell subsets (**Figures 5B**). The highest expression of CD31 was found on subsets of monocytes, while neutrophils, eosinophils, basophils and dendritic cells showed threefold lower expression (**Figures 5A**). All the CD8 T cells were CD31 positive but presented lower levels than myeloid cells, while only naive CD4 T cells showed partly positive expression (**Figure 5B**). B cells presented a heterogeneous staining pattern with lower intensity (**Figures 5B**). The highest level of CD38 expression was found on basophils and NK cells (heterogeneous), followed by myeloid dendritic cells (mDC), plasmacytoid dendritic (pDC) and B cell subsets (**Figure 5A**). CD38 expression was gradually decreased on monocyte subsets along with their maturation. Memory and effector stages of T cells lacked CD38 at steady state. However, among B cells, CD38 expression was heterogeneous, reaching high levels on naive B cells, spreading from negative to high on switched memory and mostly lacking on natural effector B cells. CD40 was expressed on all B cell subsets and absent from all other leukocyte subsets tested. The ABC units allowed for interpretable expression level evaluation, but the patterns did not differ from those observed on PE fluorescence intensity (**Data Sheet 5**).

Similar patterns of expression were observed for the proportions of positive cells within a certain subset (**Data Sheet 6**) with several differences. Nearly 100% of ILC3 were CD11b positive, while the expression levels were rather low on the basis of the median ABC. Conversely, on the basis of the median ABC (649), Tfh cells were deemed CD31 negative, while 30% of events within the Tfh cell subset were positive for CD31.

**Figure 6:**
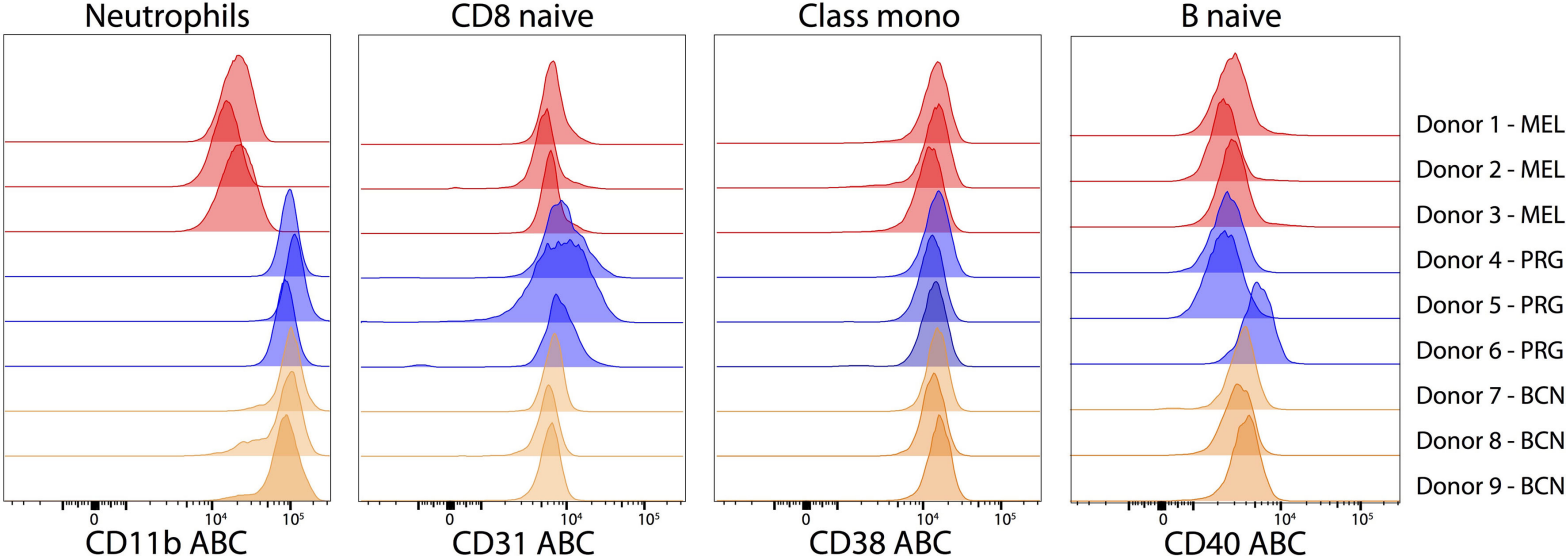
Expression variability of markers between donors and labs on selected cell subsets. Histograms of expression levels in ABC units of CD11b on neutrophils, CD31 on naive CD8^+^ T cells, CD38 on classical monocytes and CD40 on naive B cells from nine donors across three laboratories. The histogram color reflects particular laboratory.

Parallel evaluation of the procedure at three centers demonstrated highly similar results. For each PE reagent, we chose to closely examine one cell subset. The expression of CD40 on naive B cells (median ABC: 2963; IQR: 1420), CD38 on monocytes (median ABC: 14049: IQR: 1912) and CD31 on naive CD8 T cells (median ABC: 6813; IQR: 4242) was comparable across donors and sites (**Figure 6**). In contrast, CD11b (median 84100: ABC; IQR: 76212) showed site-dependent variation that was explained by the sample source. In the general CD Maps protocol, a buffy coat was used; however, the limited availability of a buffy coat forced one laboratory to use peripheral blood, which accounted for the difference (**Data Sheet 7**). The expression of CD11b on neutrophils isolated from freshly drawn peripheral blood (median ABC: 16614; IQR: 8320) is 5x lower compared to neutrophils isolated from buffy coat (median ABC: 94590; IQR: 33437).

Comparison of the three different donors analyzed in each laboratory showed highly similar results: the mean coefficient of variation (CV) reached 30% (Min: 1%; Max: 111%). The inter-laboratory mean CV was 55% (Min: 4%; Max: 137%). Full details are in the **Supplementary Table S2**.

**Figure 7:**
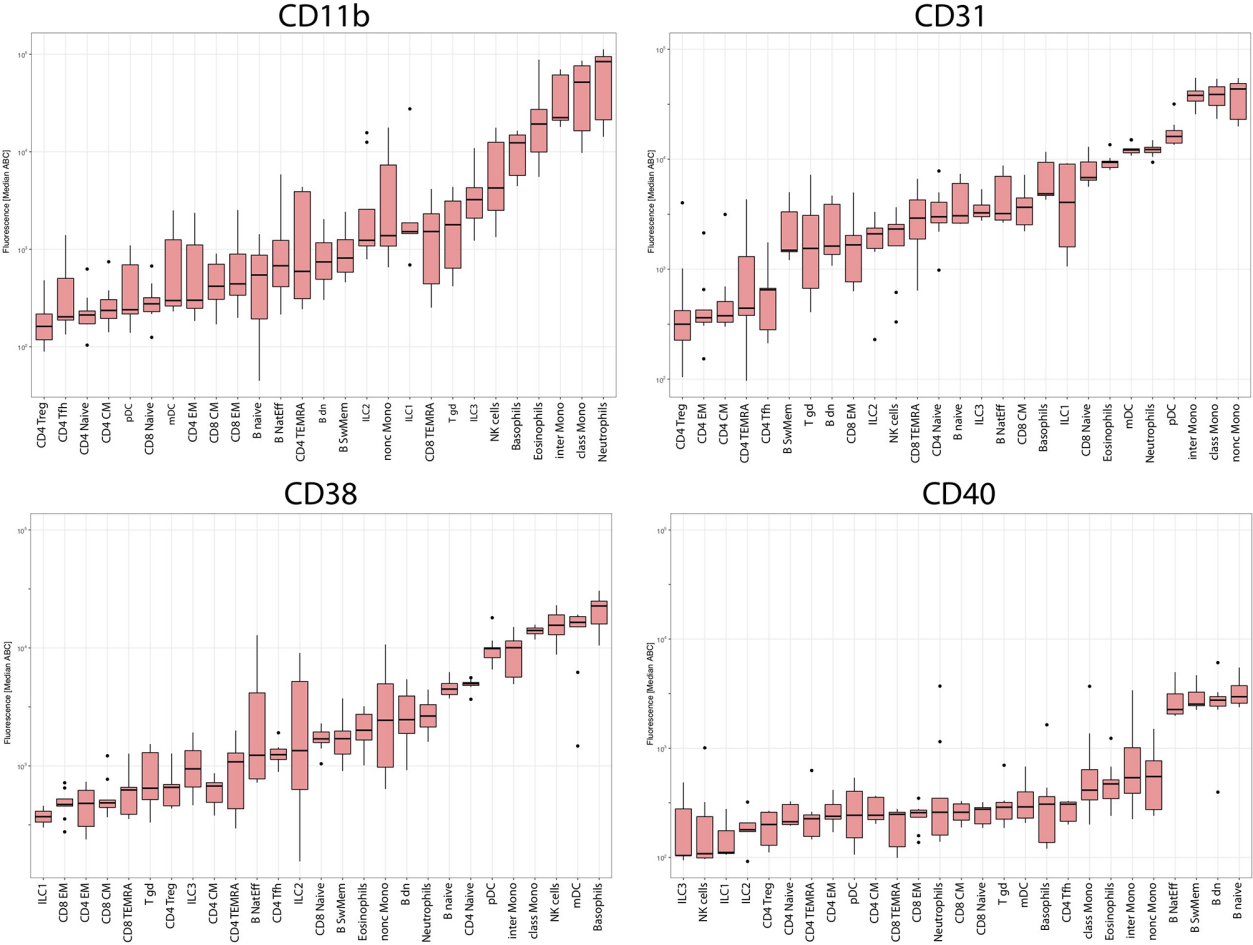
Median expression levels on all subsets in all donors. Boxplots showing expression levels in ABC units of CD11b, CD31, CD38 and CD40 on 27 leukocyte subsets ordered from cell subset with lowest expression of the particular marker to cell subset with highest expression of particular marker.

Although aggregated expression data over all subsets and all donors provided a complete picture (**Figure 7**), the histogram distribution of measured single cells allowed us to explain greater heterogeneity of the median ABC values for subsets with heterogeneous (CD11b on B cells) or bimodal (CD31 on naive CD4 T cells) expression (**Figure 5**).

To further evaluate the reproducibility of the ABC data, we compared the currently measured data to the CD Maps pilot study for the four CDs (**Supplementary Table S3**). For the cell subsets depicted in **Figure 6**, the CD11b median ABC was 1.2-fold higher in the current study on neutrophils (the same Ab clone was used but different vendors), the CD31 median ABC was 1.12-fold higher (on CD8 naive T cells; MEM-05 vs. WM59 clone using different vendors), the CD38 median ABC was 1.5-fold lower (on monocytes; using the same clone and vendor, but a titer 4 times lower), and the CD40 median ABC was 1.8-fold lower in the current study (on naive B cells; using the same clone but different vendors). Taken together, the differences in ABC expression levels between the current and CD Maps pilot study (15) were less than twofold.

### Reactivity Benchmarking of Multiple mAb Clones Targeting the Same CD Marker

**Figure 8:**
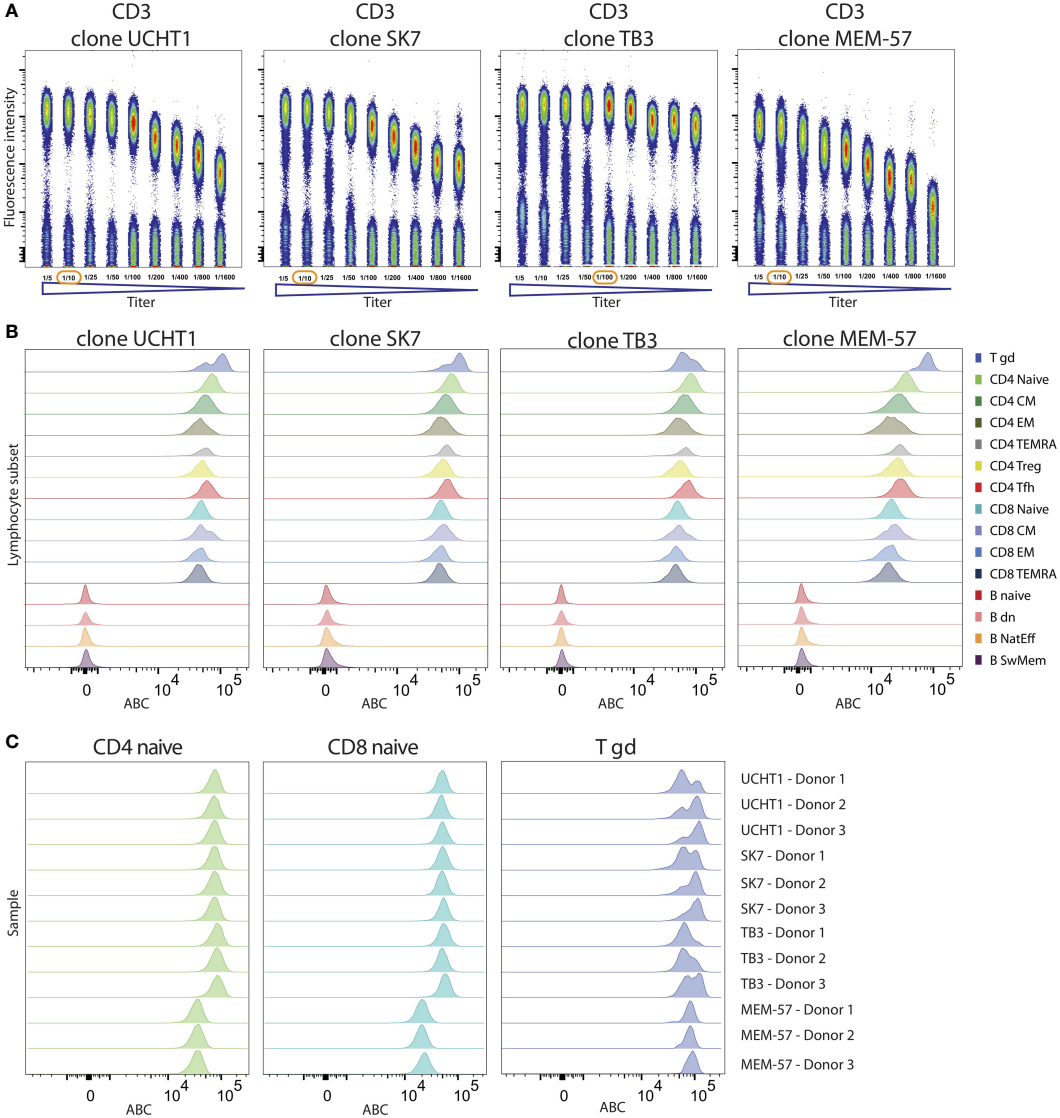
Comparison of performance of four CD3 clones. **(A)** Titration curves of four CD3 clones depicted as dotplots on gated lymphocytes with reagent titers ranging from 1/5 to 1/1600 (ratio of reagent volume within the total staining volume). The selected optimal titer is highlighted with a red circle. **(B)** Histograms of expression levels of CD3 measured with four different antibody clones are shown on B and T cell subsets. **(C)** ABC levels of four CD3 antibody clones on naive CD4, naive CD8 and TCRγδ^+^ T cells from three donors.

An important application of the CD Maps resource will be selecting appropriate mAb clones. Although all mAb clones that were validated in previous HLDA workshops will have similar expression patterns, their expression levels were not quantitatively defined, with the potential to show substantial differences. To address this issue, we applied our experimental setup with quantitative expression in ABC units to four CD3 mAb clones. These four clones were first titrated on lymphocytes, and highly different titration curves were observed, although all were reactive to the same target protein on T cells. The titration curve of the TB3 clone showed a prolonged plateau and was shorter for UCHT1 and SK7, and the expression levels of MEM-57 were decreased (**Figure 8A**).

Following optimal titer selection, all four clones were investigated in the context of the adaptive cell tube. As expected, all four clones were specifically reactive to all T cell subsets and did not stain any of the B cells (**Figure 8B**). However, within the T cell subsets, we observed quantitative differences depending on the mAb clone. T cell receptor (TCR) γδ^+^ T cells showed higher expression levels of CD3, a finding that was consistently observed for all four clones (from 71620 to 85479). Three clones (SK7, UCHT1 and TB3) showed similar levels of CD3 expression on naive CD4 T cells (71079, 71419, 80876 ABC units) and naive CD8 T cells (47735, 49610, 51909 ABC units, respectively). By contrast, the MEM-57 clone yielded lower CD3 expression levels for both CD4 (33540 ABC) and CD8 (20745 ABC) T cells (**Figure 8C**). These results suggest that the CD3 epitope recognized by MEM-57 is less accessible on TCRαβ^+^ T cells, resulting in lower measured ABC. Thus, the MEM-57 clone enables the distinction of TCRαβ^+^ from TCRγδ^+^ T cells based on the CD3 expression intensity. However, the titration characteristics make MEM-57 suboptimal for CD3 expression quantification. The surface expression levels of CD3 on T cell subsets can be reproducibly quantified using three independently developed CD3 clones (SK7, UCHT1 and TB3) across measured donors.

## Discussion

Here, we demonstrate a high-throughput procedure for the expression profiling of surface antigens on 27 leukocyte subsets that is standardized across global laboratories for accurate quantification. This optimized procedure overcomes previous limitations observed in the CD Maps pilot project mapping the expression of CD1 to CD100 (dynamic web resource at hcdm.org) as follows (15): 1) Expansion of backbone markers enabled the distinction of additional T cell subsets (Treg and Tfh) and innate lymphoid cells (ILC-1, ILC-2 and ILC-3); 2) a universal titration procedure was adopted for each PE marker, improving the quantitation and 3) excitation of PE from the 561 nm laser led to increased sensitivity to markers with low expression levels. The procedure presented here will now facilitate re-evaluation of all approved CD markers (CD Maps) and validation of new mAbs for CD marker designation in HLDA workshops.

The same surface markers were used to define the leukocyte subsets that were previously defined in the CD Maps pilot project except for IgM. Thus, the definition for naive and natural effector B cells was slightly different. Because all naive B cells (CD27^neg^IgD^+^) express IgD, these cells and natural effector B cells (CD27^+^IgD^+^) were hardly affected (36, 37). The current approach will have left IgM-only memory B cells (CD27^+^IgM^+^IgD^neg^) in the CD27^neg^IgD^neg^ population, making this a mixture of unswitched and switched memory B cells (38).

The definition of DC subsets relies on excluding CD3^+^, CD19^+^, CD14^+^ and CD16^+^ cells, high HLA-DR expression and subdivision of CD123^+^ (pDCs) and CD11c^+^ (mDCs) (39, 40), while additional markers can be used to identify and separate myeloid DCs into two finer subsets (conventionalDC1: CD141, CD370 and conventionalDC2: CD1c, CD301) (39, 41).

For T cell subsets, we used CD45RA and CD27 to resolve the naive (CD45RA^+^CD27^+^) and central memory (CD45RA^neg^CD27^+^) stages from the effector memory (CD45RA^neg^CD27^neg^) stage, in accordance with the definition strategy used by the EuroFlow group for human primary immunodeficiency (42–44). The advantage of this approach is that CD27 can be used for subsets of B cells and T cells, making the antibody backbone simpler. Furthermore, sample processing and antibody clone selection was reported as a source of false negative staining with CD62L and CD197 respectively (6). Although CD62L and CD197 delineate the same cells as naive cells (44), they delineate a small but consistent population of transitional effectors (CD27^+^CD62L^neg^CD197^neg^) (45), a population that is blended to central memory in our dataset. Effector memory (EM) and terminal effector memory (TEMRA) stages contain further subsets of early, intermediate and late stages defined by CD28 expression (42) and correlate with the chronic carrier status of Cytomegalovirus and Epstein-Barr virus (46, 47). Because expanding the knowledge of T cell subsets by polychromatic cytometry has led to identification of new functional subsets (48), subsets defined by checkpoint inhibitors (49) or tissue-specific subsets (28) at a fast pace, our simplified subset definitions may yield heterogeneous expression signals on some subsets containing finer subtypes, but the general description will be true and useful nonetheless.

Detection of the PE signal from excitation with the 561 nm laser in the current study improved signal sensitivity because of decreased autofluorescence of leukocytes and reduced the data spread from decreased spillover of the FITC reagent, as expected (50). These two effects combined led to a decreased background on myeloid subsets (384 ABC units) and allowed for more reliable detection of low-expressing markers than we had previously observed in the CD Maps pilot study (background of 1026 ABC units) (15).

We have addressed an important issue of proper titration (5) by building a uniform titration protocol, where we added cell lines representing leukocyte types and peripheral blood cells to allow for proper titer estimation. Our approach was specifically designed for mAbs submitted for 11^th^ HLDA workshop and the CDmaps2 project. Although most antigens targeted in this study are expressed at sufficient levels on peripheral blood cells, other known markers such as activation markers (CD25, CD54, CD69 or CD80) are expressed at very low levels or small subsets but are expressed on the selected cell lines. Other cell lines may be appropriate for titration, when the surface markers investigated will include markers expressed solely on stem cells, platelets, or endothelial cells. Because nonspecific staining is a problematic aspect of antibody reagent binding at given concentrations, we used a mouse cell line to ensure the presence of a negative cell type that can be easily evaluated. We selected titers that balance the following two objectives: 1) mAb staining was near saturation to provide accurate ABC measurement and achieve reproducibility; 2) minimal background fluorescence on defined negative cells allow the specific quantification of target molecules with low levels of expression. The titration curves of the four CD3 clones illustrated that they depend highly on whether the mAb clone titer is an ideal balance of these two objectives, demonstrating the need to select the right mAb clone for the experimental objective.

Large-scale expression profiling studies involve an extensive experimental setup, with expansion of the backbone from 7 to 10-11 colors in our study, resulting in added experimental complexity. To optimize the sample preparation procedure, a dried antibody backbone mixture was custom produced in 96-well plates. This allowed fast and robust sample staining of batches of 96-well plates and high interlaboratory data comparability due to limited pipetting errors.

Another challenging aspect of CD Maps resource is the handling of large datasets. This issue was addressed using R-project scripts presented *via* the Shinny interface to annotate (clone names, titers, and manufacturers) the acquired measured FCS files.

To accurately quantify surface molecule expression and visualize intercell and interindividual variation, the technical variability must be minimized. Here, we build on previous expertise obtained from the CD Maps pilot study (15) with further refinement of titration and PE excitation. Compared with the CD Maps pilot project, we reached similar quantitative results for CD11b, CD31, CD38, and CD40. Comparable results were achieved despite using specimens from different donors, acquisition 5 years later using new instrumentation, different staffing and PE reagents obtained from different vendors (3 of four different) highlighting the robustness of the standardization procedure. This finding agrees with the long-term experience of the EuroFlow consortium, where reproducible signal intensity measurement is achievable using thorough standardization (51) and is exploited for quality assessment purposes applied worldwide (52). Thus, the EuroFlow consortium can use CD marker reagents from different vendors with comparable intensity measurements (18). Of the markers tested here, CD3 and CD38 are currently used in EuroFlow QA.

However, preanalytical sample handling procedures can alter the expression level of particular surface molecules on granulocytes (53). Here, we observed a 4.5-fold increase in CD11b ABC after processing buffy coat samples compared with freshly drawn peripheral blood cells; additionally, CD11b can increase with activation or with density gradient isolation (54). Lymphocyte subsets generally show higher stability of expression than myeloid subsets with prolonged storage; however, specimens measured within 24 h after the blood draw maintain stable expression (55).

Evaluating the surface expression and reagent performance at the level of defined subsets provides an opportunity to reach reproducible readouts for markers with complex expression profiles (e.g., uniform CD38 positivity on monocytes but heterogeneous expression on unselected leukocytes) (5). Furthermore, the comparison between four CD3 clones demonstrates that quantitative differences in the ABC exist among clones, in which three CD3 clones reach very similar ABC values, while one clone consistently differs on TCRαβ+ subsets. Thus, extension of the CD Maps project from one representative reagent against each CD to multiple (all available) reagents is warranted, providing reactivity benchmarking. Meaningful ABC evaluations must, however, be performed on correctly titrated antibody conjugates.

In conclusion, we have developed and optimized a method for reproducible, high throughput evaluation of CD marker expression on 27 human peripheral blood subsets. Its primary use is for the completion of the CD Maps project, aiming to quantitatively profile the expression of all surface molecules assigned with CD nomenclature within all 10 historical HLDA workshops. Furthermore, this method will be applied to evaluate reactivity of all newly submitted reagents within the current 11^th^ HLDA workshop. The robust and standardized nature of our procedure will enable benchmarking the reactivity of PE-conjugated antibody reagents (new or established). These implementations will provide the CD Maps resource managed by HCDM.org with representative reagents to all CD markers, and it will catalog all submitted reagents against that CD target, thereby providing the community with an experimental benchmarking platform in a structured and searchable format.

## Data Availability Statement

The original contributions presented in the study are included in the **Supplementary Material**. The original fcs files are deposited on the HCDM website (https://www.hcdm.org/index.php/2016-12-06-21-38-08/cdmaps-data-repository. Further inquiries can be directed to the corresponding author.

## Ethics Statement

The studies involving human participants were reviewed and approved by Human Ethics Committee of Monash University, Ethics Committee of Motol University Hospital, Ethics Committee of the University of Barcelona. Written informed consent for participation was not required for this study in accordance with the national legislation and the institutional requirements.

## Author Contributions

Contribution: TK, PE, and MZ conceptualized the study and designed the experiments. DK, JP-O, PA, and JF performed the experimental work. DK, TK, and JP-O performed data analysis. KF built the online web tools. DK, TK, and MZ wrote the paper. All authors commented on draft versions and approved the final manuscript.

## Funding

The work was financially supported by the International Union of Immunological Societies (IUIS), projects NU20-05-00282 of the Czech Republic Ministry of Health to TK, Australian National Health and Medical Research Council (NHMRC) Fellowship GNT1117687 to MZ, SAF2015-69829 from Ministerio Ciencia e Innovación, Spain to PE.

## Conflict of Interest

BioLegend and Exbio kindly donated reagents used in this study.

The authors declare that the research was conducted in the absence of any commercial or financial relationships that could be construed as a potential conflict of interest.​​​​​​​​​​​​​​​​​​​​

## Publisher’s Note

All claims expressed in this article are solely those of the authors and do not necessarily represent those of their affiliated organizations, or those of the publisher, the editors and the reviewers. Any product that may be evaluated in this article, or claim that may be made by its manufacturer, is not guaranteed or endorsed by the publisher.

## Abbreviations

ABC, Antibody binding capacity; CV, Coefficient of variation; EMT, Experiment Master Table; FMO, fluorescence minus one; HCDM, Human Cell Differentiation Molecules; HLDA, Human Leukocyte Differentiation Antigen; HTS, High Throughput Sampler; ILC, innate lymphoid cells; IQR, interquartile range; mAb, monoclonal antibody; mDC, myeloid dendritic cells; pDC, plasmacytoid dendritic cells; PE, Phycoerythrin; RT, room temperature; SOP, Standard Operating Procedure; TCR, T cell receptor; Tfh, T follicular helper cells; Treg, T regulatory cells.
