# Supplementary material for: Standardization of Workflow and Flow Cytometry Panels for Quantitative Expression Profiling of Surface Antigens on Blood Leukocyte Subsets: An HCDM CDMaps Initiative
Source: Front Immunol. 2022 Feb 11;13:827898. doi: 10.3389/fimmu.2022.827898 (PMC8874145; doi:10.3389/fimmu.2022.827898)
Supplement: Supplementary file 1 [file DataSheet_1.pdf]

L210616_QC4_repeat_Innate

HLDA11 workshop + CDmaps2

Material: Buffy coat
amount of buffy coat depends on how many markers you are going to measure

Solutions:
the amount of solutions you need depends on the amount of buffy coat
4% dextran solution (in 0.9% NaCl): 0,9g NaCl, 4g dextran in 100 mL bidestilled water.
PBS without Ca and Mg plus 2 mM EDTA (PBS with EDTA)
PBS+0.09% of NaN3+0.5% BSA + 20% rabbit serum (=rabbit serum solution)
Exbio Excelyse Easy dilute 10x in dH2O (=lysis solution)

Blood extraction work in a tube

1. Dilute buffy coat 1:5 with sterile PBS with EDTA
2. Mix 4% dextran solution with the diluted BC in a 50 mL tube 1:1
3. Mix (up and down) and let erythrocytes sediment for 30 min.
4. Collect the supernatant carefully (avoid to take erythrocytes) and centrifuge (670g, 5 min, RT)
5. Remove the supernatant.
6. Adjust WBC with viability stain, final concentration 50M/ml
7. For dilution use rabbit serum solution
8. For staining you need 2M (=40ul) of live cells/well

Staining work in 96-deep well plate (2ml)

1. Where needed add BSA solution into wells
2. Add PE markers into the 96 deep well plate
3. Add 2M (40ul) of cells into well, gentle vortex (use your hand to soften the vibration)
   50ul final volume of the test (cell suspension + marker +human serum)
4. incubate (30min, RT, dark)
5. prepare premix of BioLegend backbone reagents according to the table
6. add drop-in mix into dried reagents. Add rabbit serum solution into the premix: into Innate 20ul, into Lympho 23.8ul
7. transfer the backbone premix (25ul) into wells
8. incubate (30min, RT, dark)
9. add lysis solution (1.5ml of 10x diluted into 75ul of cell suspension), gentle vortex or mix with the pipette
10. incubation 10min, RT, dark
11. spin (670g, 5min, RT)
12. remove the supernatant (dump to sink filled with ice)
13. add 170ul PBS
14. keep overnight in the fridge (dark)

Experiment preparation in DIVA

1. Experiment name has to perfectly match to name of PE markers.xls
2. Highlight all wells in the plate, click add specimen
3. Label the channels
4. Set properly the HTS (sample volume + events to be recorded)

Acquisition work in 96-well V bottom plate

1. tranfer unstained cell suspension into a FACS tube
2. transfer stained cell suspension into 96well V-bottom plate (300ul)
3. use unstained control for proper setting of FSC- and SSC voltage and FSC-threshold
4. control of proper FSC-threshold setup: plot FSC vs CD45 - did I cut some cells? If yes, descrease the FSC-threshold
5. acquire the samples from 96 well plate
6. Resolve Quantibrite beads in 500ul of BSA solution just before acquisition

HTS setup - can be adjusted according to your experience
sample flow rate 3 ul/sec
sample volume 160ul mixing volume 100ul mixing speed 200ul/sec
number of mixes 2
wash volume 400ul

abort rate: do not exceed 10% of recorded events

Important points for the fcs files conversion
Experiment name has to perfectly match with PE marker table name
Plate name has to perfectly match with the PLATE column in PE marker table

mix

fluor	TARGET	CLONE	VENDOR	CAT_NO	PANEL	AB_VOLUME	PLATE_+15%
BV421	CD127	A019D5	BioLegend	351310	Innate_dried_reagents	1.25	24.4375
Pacific Orange	CD45	2D1	Exbio	ED7094-1	Innate_dried_reagents	NA	NA
BV605	CRTH2	BM16	BioLegend	350122	Innate_dried_reagents	2.00	39.1000
BV711	CD56	HCD56	BioLegend	318336	Innate_dried_reagents	1.25	24.4375
FITC	CD117	104D2	Exbio	ED7174-1	Innate_dried_reagents	NA	NA
PE-DyLight 594	CD3	UCHT1	Exbio	ED7248-1	Innate_dried_reagents	NA	NA
PE-DyLight 594	CD19	LT19	Exbio	ED7227-1	Innate_dried_reagents	NA	NA
PerCP-Cy5,5	CD14	NA	Exbio	ED7507-1	Innate_dried_reagents	NA	NA
PE-Cy™7	CD11c	BU15	Exbio	ED7500-1	Innate_dried_reagents	NA	NA
APC	CD123	6H6	Exbio	ED7246-1	Innate_dried_reagents	NA	NA
Alexa Fluor® 700	HLA-DR	L243	Exbio	ED7238-1	Innate_dried_reagents	NA	NA
APC-Cy™7	CD16	3G8	Exbio	ED7516-1	Innate_dried_reagents	NA	NA

PLATE:

well_row	1	2	3	4	5	6	7	8	9	10	11	12
A	—	—	—	—	—	—	—	—	—	—	—	—
B	—	—	—	—	—	—	—	—	—	—	—	—
C	—	—	—	—	—	—	—	—	—	—	—	—
D	PECAM1<br>CD31<br>MEM-05<br>Exbio<br>5 + 5 ul PBS	CD38<br>CD38<br>HIT2<br>Exbio<br>5 + 5 ul PBS	TNFRSF5<br>CD40<br>5C3<br>BioLegend<br>1 + 9 ul PBS	ITGAM<br>CD11b<br>ICRF44<br>BioLegend<br>5 (10_dil) + 5 ul PBS	FMO<br>FMO<br>NA<br>NA<br>0 ul	unstained<br>unstained<br>NA<br>NA<br>0 ul	—	—	—	—	—	Quantibrite<br>Quantibrite<br>NA<br>NA<br>0 ul
E	PECAM1<br>CD31<br>MEM-05<br>Exbio<br>5 + 5 ul PBS	CD38<br>CD38<br>HIT2<br>Exbio<br>5 + 5 ul PBS	TNFRSF5<br>CD40<br>5C3<br>BioLegend<br>1 + 9 ul PBS	ITGAM<br>CD11b<br>ICRF44<br>BioLegend<br>5 (10_dil) + 5 ul PBS	FMO<br>FMO<br>NA<br>NA<br>0 ul	—	—	—	—	—	—	—
F	PECAM1<br>CD31<br>MEM-05<br>Exbio<br>5 + 5 ul PBS	CD38<br>CD38<br>HIT2<br>Exbio<br>5 + 5 ul PBS	TNFRSF5<br>CD40<br>5C3<br>BioLegend<br>1 + 9 ul PBS	ITGAM<br>CD11b<br>ICRF44<br>BioLegend<br>5 (10_dil) + 5 ul PBS	FMO<br>FMO<br>NA<br>NA<br>0 ul	—	—	—	—	—	—	—
G	—	—	—	—	—	—	—	—	—	—	—	—
H	—	—	—	—	—	—	—	—	—	—	—	—
