# Supplementary material for: Standardization of Workflow and Flow Cytometry Panels for Quantitative Expression Profiling of Surface Antigens on Blood Leukocyte Subsets: An HCDM CDMaps Initiative
Source: Front Immunol. 2022 Feb 11;13:827898. doi: 10.3389/fimmu.2022.827898 (PMC8874145; doi:10.3389/fimmu.2022.827898)
Supplement: Supplementary file 2 [file DataSheet_2.pdf]

Name	...	...	maps_CD	maps_TARGET	maps_CLONE	maps_VENDOR	maps_CAT_NO	maps_AB_VOLUME	maps_PLATE	maps_WELL_ID	maps_MATE...	maps_PANEL	maps_EXPERIMENT	maps_INST	maps_OPERATOR	maps_PROJECT	maps_well_col	maps_well_row
PECAM1_PLATE_D01_BC17.fcs		...	CD31	PECAM1	MEM-05	Exbio	1P-273-T100	5	PLATE	D01	BC17	Innate_dried_reagents	L210616_QC4_repeat_Innate	PRG	DK	QC	1	D
CD38_PLATE_D02_BC17.fcs		...	CD38	CD38	HIT2	Exbio	1P-366-T100	5	PLATE	D02	BC17	Innate_dried_reagents	L210616_QC4_repeat_Innate	PRG	DK	QC	2	D
TNFRSF5_PLATE_D03_BC17.fcs		...	CD40	TNFRSF5	5C3	BioLegend	334308	1	PLATE	D03	BC17	Innate_dried_reagents	L210616_QC4_repeat_Innate	PRG	DK	QC	3	D
ITGAM_PLATE_D04_BC17.fcs		...	CD11b	ITGAM	ICRF44	BioLegend	301306	0.5	PLATE	D04	BC17	Innate_dried_reagents	L210616_QC4_repeat_Innate	PRG	DK	QC	4	D
FMO_PLATE_D05_BC17.fcs		...	FMO	FMO	NA	NA	NA	0	PLATE	D05	BC17	Innate_dried_reagents	L210616_QC4_repeat_Innate	PRG	DK	QC	5	D
PECAM1_PLATE_E01_BC18.fcs		...	CD31	PECAM1	MEM-05	Exbio	1P-273-T100	5	PLATE	E01	BC18	Innate_dried_reagents	L210616_QC4_repeat_Innate	PRG	DK	QC	1	E
CD38_PLATE_E02_BC18.fcs		...	CD38	CD38	HIT2	Exbio	1P-366-T100	5	PLATE	E02	BC18	Innate_dried_reagents	L210616_QC4_repeat_Innate	PRG	DK	QC	2	E
TNFRSF5_PLATE_E03_BC18.fcs		...	CD40	TNFRSF5	5C3	BioLegend	334308	1	PLATE	E03	BC18	Innate_dried_reagents	L210616_QC4_repeat_Innate	PRG	DK	QC	3	E
ITGAM_PLATE_E04_BC18.fcs		...	CD11b	ITGAM	ICRF44	BioLegend	301306	0.5	PLATE	E04	BC18	Innate_dried_reagents	L210616_QC4_repeat_Innate	PRG	DK	QC	4	E
FMO_PLATE_E05_BC18.fcs		...	FMO	FMO	NA	NA	NA	0	PLATE	E05	BC18	Innate_dried_reagents	L210616_QC4_repeat_Innate	PRG	DK	QC	5	E
PECAM1_PLATE_F01_BC19.fcs		...	CD31	PECAM1	MEM-05	Exbio	1P-273-T100	5	PLATE	F01	BC19	Innate_dried_reagents	L210616_QC4_repeat_Innate	PRG	DK	QC	1	F
CD38_PLATE_F02_BC19.fcs		...	CD38	CD38	HIT2	Exbio	1P-366-T100	5	PLATE	F02	BC19	Innate_dried_reagents	L210616_QC4_repeat_Innate	PRG	DK	QC	2	F
TNFRSF5_PLATE_F03_BC19.fcs		...	CD40	TNFRSF5	5C3	BioLegend	334308	1	PLATE	F03	BC19	Innate_dried_reagents	L210616_QC4_repeat_Innate	PRG	DK	QC	3	F
ITGAM_PLATE_F04_BC19.fcs		...	CD11b	ITGAM	ICRF44	BioLegend	301306	0.5	PLATE	F04	BC19	Innate_dried_reagents	L210616_QC4_repeat_Innate	PRG	DK	QC	4	F
FMO_PLATE_F05_BC19.fcs		...	FMO	FMO	NA	NA	NA	0	PLATE	F05	BC19	Innate_dried_reagents	L210616_QC4_repeat_Innate	PRG	DK	QC	5	F
Quantibrite_PLATE_A12_NA.fcs		...	Quantibrite	Quantibrite	NA	NA	NA	0	PLATE	A12	NA	Innate_dried_reagents	L210616_QC4_repeat_Innate	PRG	DK	QC	12	A
