# Supplementary material for: Standardization of Workflow and Flow Cytometry Panels for Quantitative Expression Profiling of Surface Antigens on Blood Leukocyte Subsets: An HCDM CDMaps Initiative
Source: Front Immunol. 2022 Feb 11;13:827898. doi: 10.3389/fimmu.2022.827898 (PMC8874145; doi:10.3389/fimmu.2022.827898)
Supplement: Supplementary file 3 [file DataSheet_3.pdf]

Data Sheet 3.

Supplementary titration data

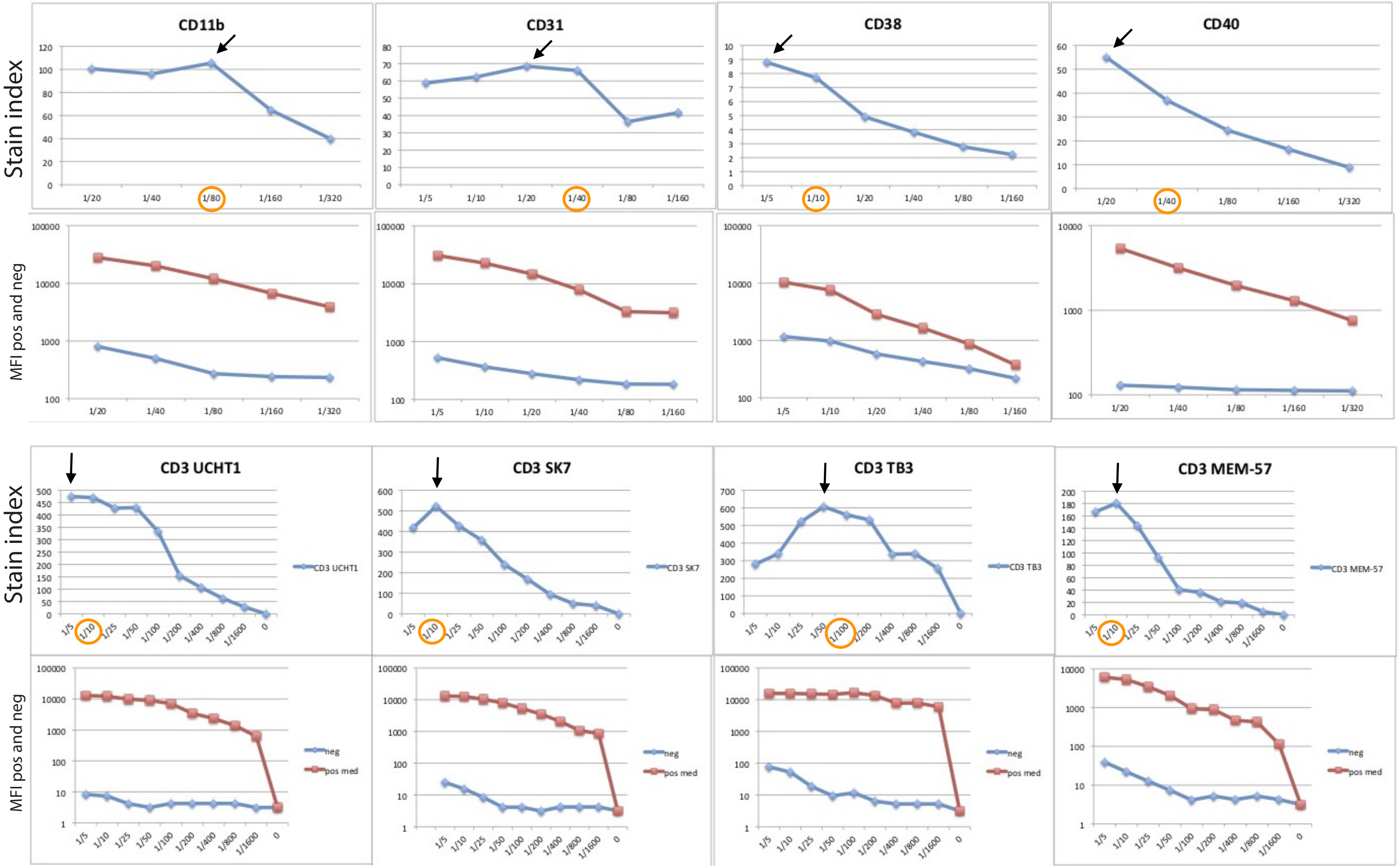

Upper graphs show Stain index, calulated according to modification by Telford et al, Cytometry A, 2009, 10.1002/cyto.a.20790
Lower graphs show median of positive population (in red) and median of negative population (in blue).
Orange circles show titers chosen on CDMaps criteria, arrows show the highest points of the Stain index curve.
