# Supplementary material for: Standardization of Workflow and Flow Cytometry Panels for Quantitative Expression Profiling of Surface Antigens on Blood Leukocyte Subsets: An HCDM CDMaps Initiative
Source: Front Immunol. 2022 Feb 11;13:827898. doi: 10.3389/fimmu.2022.827898 (PMC8874145; doi:10.3389/fimmu.2022.827898)

Data sheet 4. Titration plots for CD31 and CD38 for electronically gated Granulocytes (left plot), Monocytes (middle plot) and Lymphocytes (right plot). The x-axis represents the dilution of mAbs including fluorescence minus one (FMO) control, the y-axis represents the intensity in PE channel. Mouse cell line (300.19) was considered as universal negative cell population. Selected titer is indicated with an arrow.

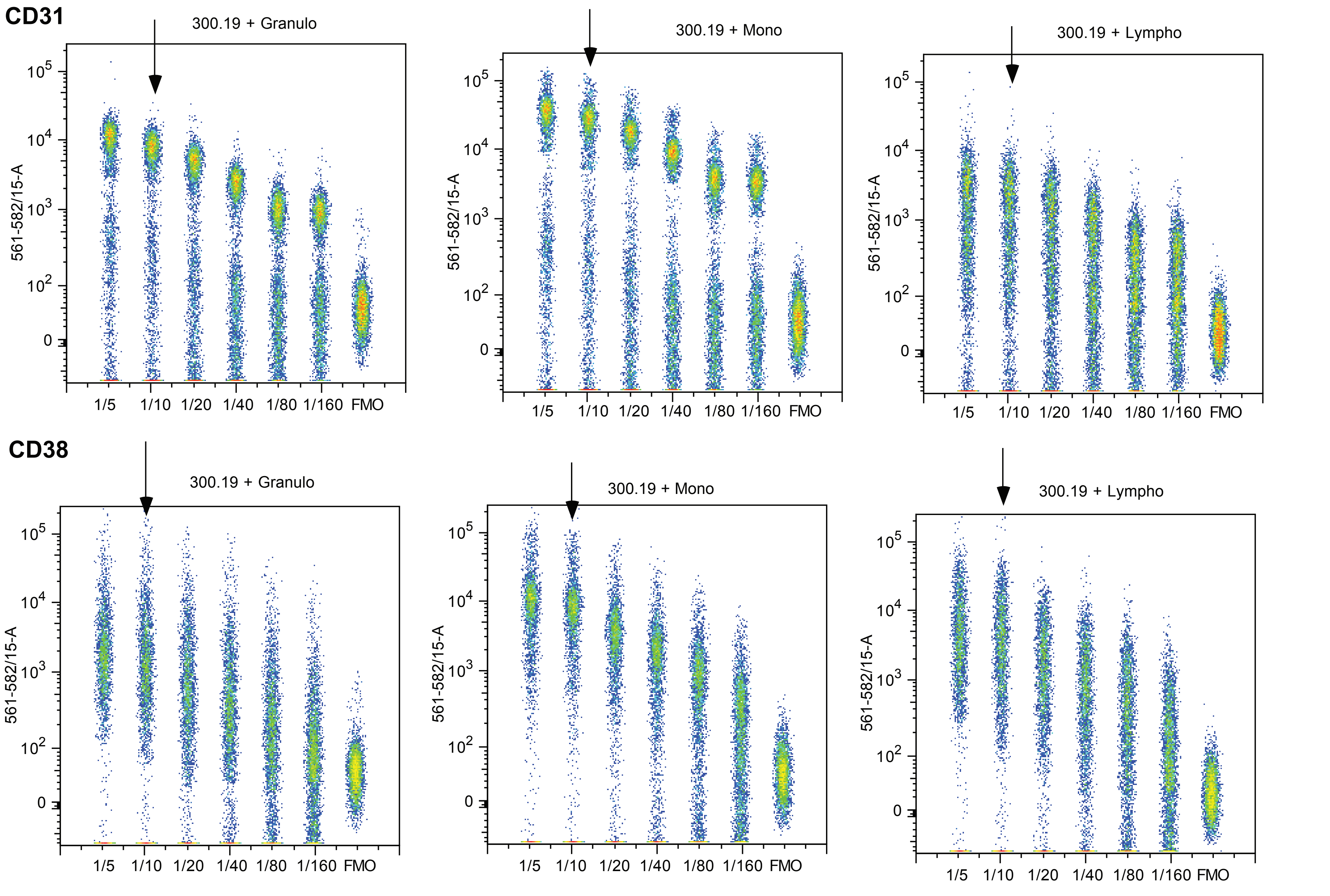

Supplement: Supplementary file 4 [file DataSheet_4.pdf]
