# Supplementary material for: Standardization of Workflow and Flow Cytometry Panels for Quantitative Expression Profiling of Surface Antigens on Blood Leukocyte Subsets: An HCDM CDMaps Initiative
Source: Front Immunol. 2022 Feb 11;13:827898. doi: 10.3389/fimmu.2022.827898 (PMC8874145; doi:10.3389/fimmu.2022.827898)
Supplement: Supplementary file 5 [file DataSheet_5.pdf]

A

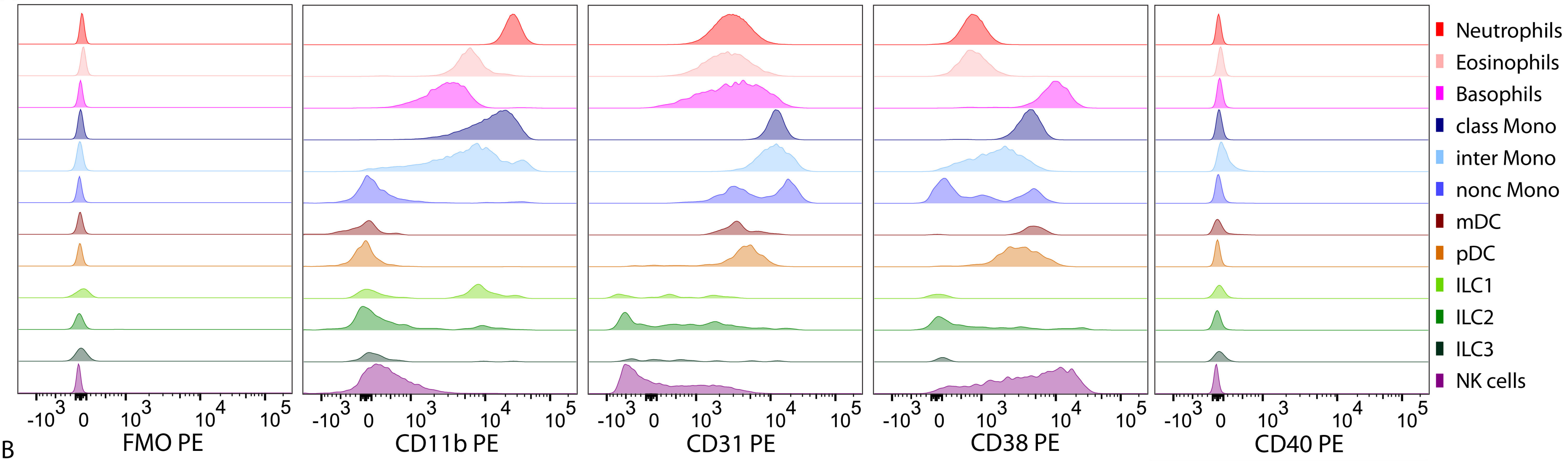


B

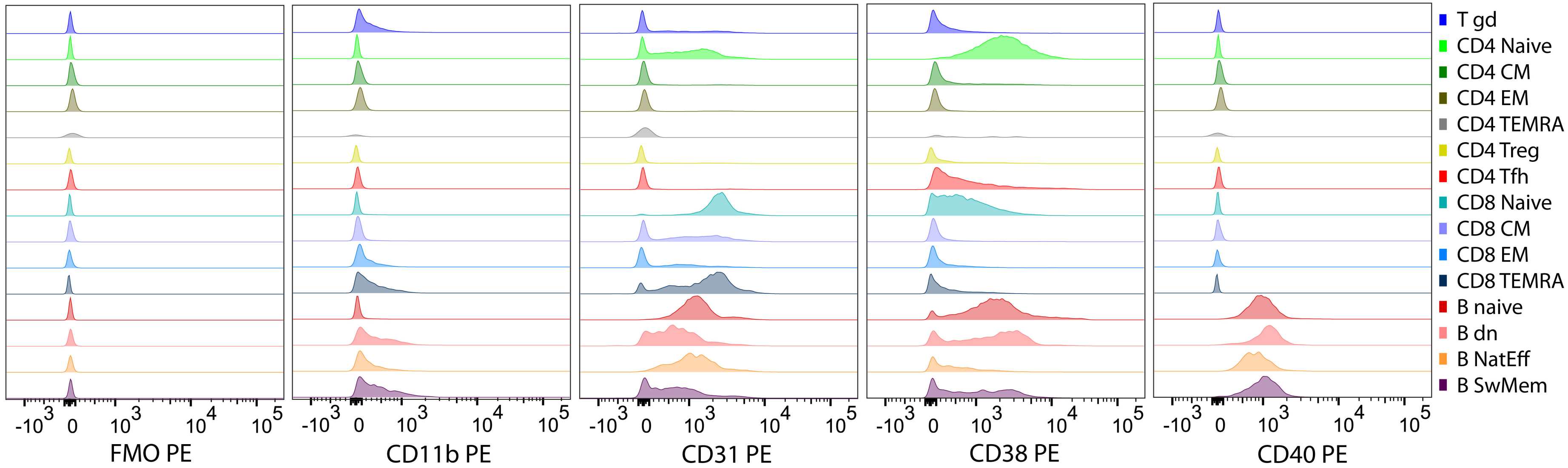


**Data Sheet 5.**

(A) Histograms of expression levels of CD11b, CD31, CD38 and CD40 are shown on innate leukocytes and (B) on lymphocytes of adaptive immunity in comparison to fluorescence minus one (FMO) controls. Expression is showed as measured with phycoerythrin conjugate.
