# Supplementary material for: Standardization of Workflow and Flow Cytometry Panels for Quantitative Expression Profiling of Surface Antigens on Blood Leukocyte Subsets: An HCDM CDMaps Initiative
Source: Front Immunol. 2022 Feb 11;13:827898. doi: 10.3389/fimmu.2022.827898 (PMC8874145; doi:10.3389/fimmu.2022.827898)

Data Sheet 6.

Boxplots showing proportion of CD11b, CD31, CD38 and CD40 positive cells within particular cell subset.
Altogether 27 leukocyte subsets are shown ordered from cell subset with lowest proportion of positive cells to cell subset with highest proportion of positive cells.

CD11b

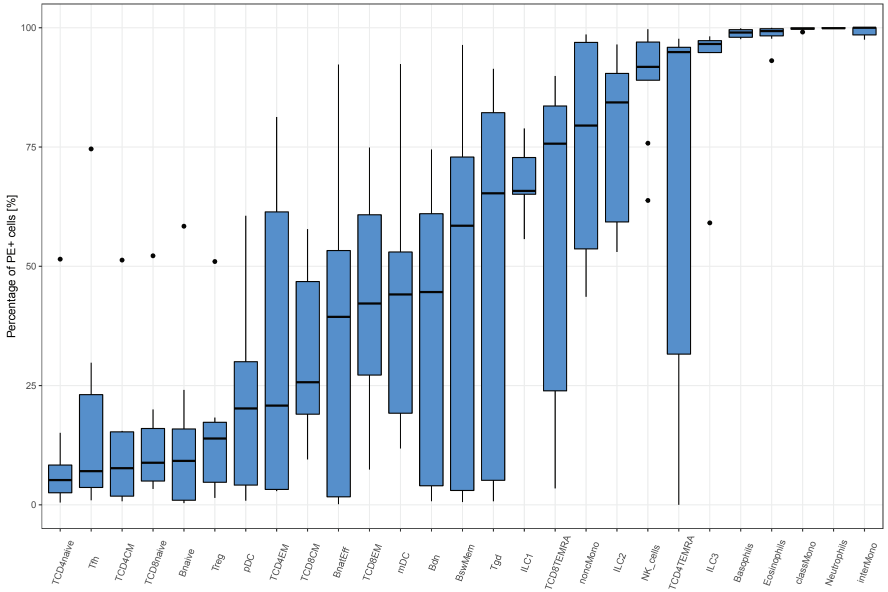

CD31

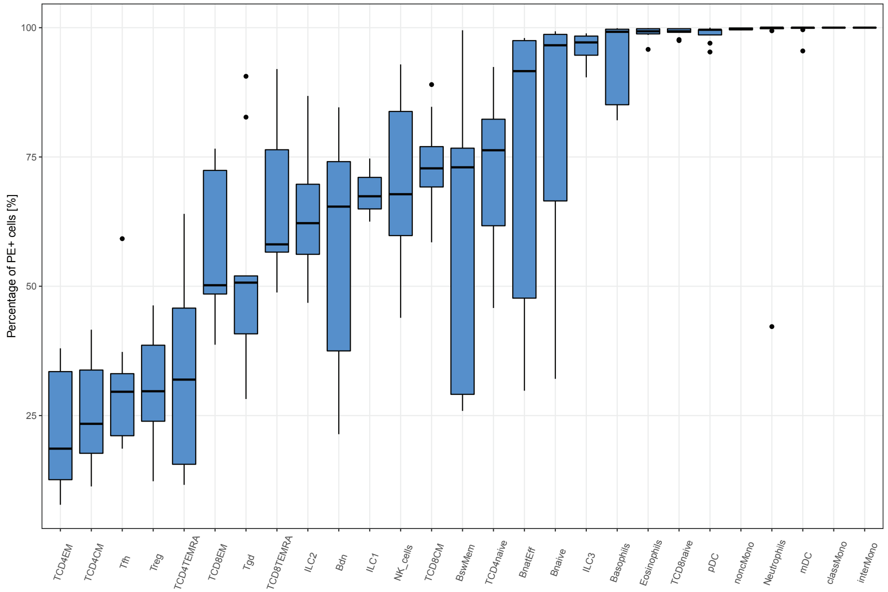

CD38

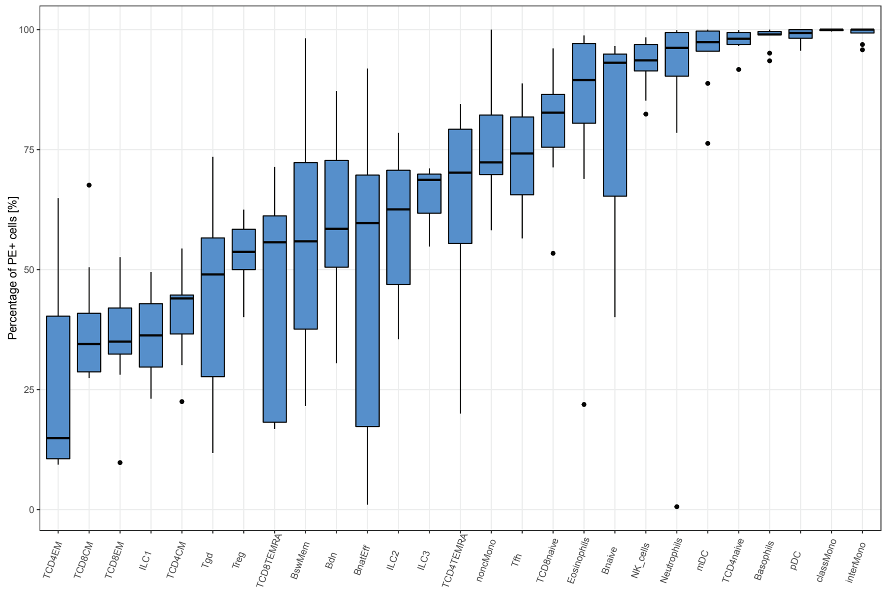

CD40

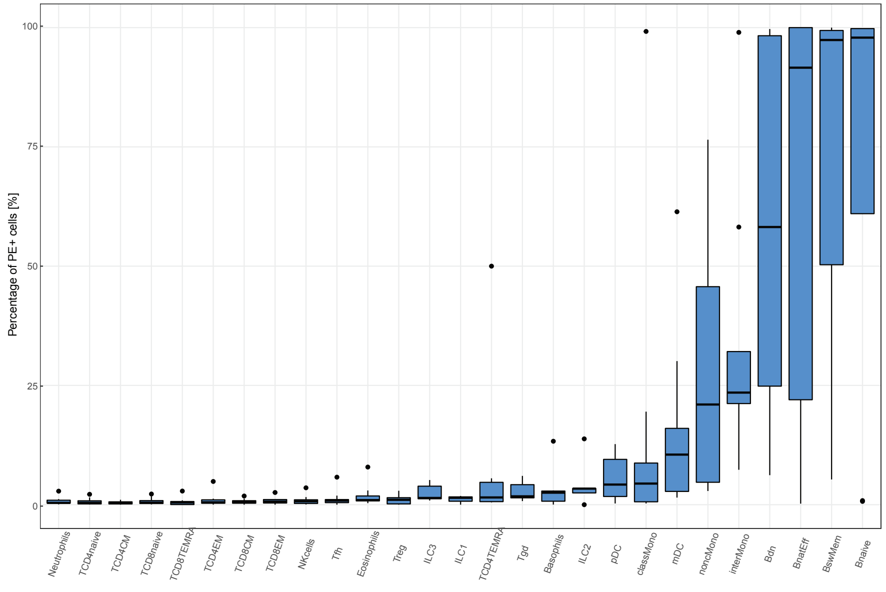

Supplement: Supplementary file 6 [file DataSheet_6.pdf]
