# Supplementary material for: Standardization of Workflow and Flow Cytometry Panels for Quantitative Expression Profiling of Surface Antigens on Blood Leukocyte Subsets: An HCDM CDMaps Initiative
Source: Front Immunol. 2022 Feb 11;13:827898. doi: 10.3389/fimmu.2022.827898 (PMC8874145; doi:10.3389/fimmu.2022.827898)

Data Sheet 7.

Neutrophils of triplets of healthy donors are separated from peripheral blood in Melbourne, or Prague or separated from buffy coats in Prague show sample type specific increase in the CD11b expression level.

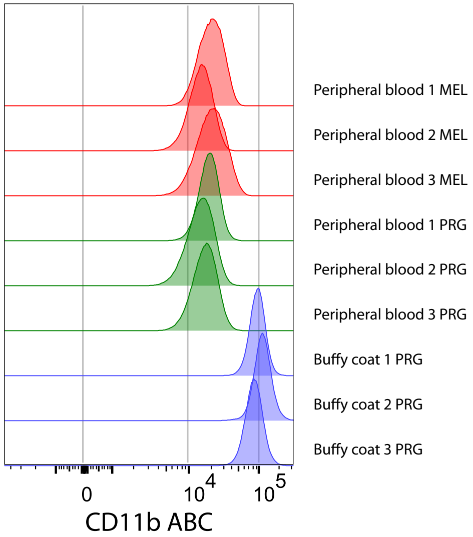

Supplement: Supplementary file 7 [file DataSheet_7.pdf]
